# Diagnostic efficiency of exome-based sequencing in pediatric patients with epilepsy

**DOI:** 10.3389/fgene.2024.1496411

**Published:** 2025-01-21

**Authors:** Huafang Zou, Qian Zhang, Jianxiang Liao, Dongfang Zou, Zhanqi Hu, Bing Li, Li Chen, Jialun Wen, Xia Zhao, Victor Wei Zhang, Dezhi Cao

**Affiliations:** ^1^ Department of Neurology, Shenzhen Children’s Hospital, Shenzhen, China; ^2^ Division of Epilepsy Surgery, Shenzhen Children’s Hospital, Shenzhen, China; ^3^ Department of Genomic Medicine, AmCare Genomics Lab, Guangzhou, China

**Keywords:** epilepsy, seizure, next-generation sequencing, whole exome sequencing, genetic diagnosis

## Abstract

**Objective:**

Epilepsy, a prevalent neurological disorder, has multifaceted etiologies. Next-generation sequencing (NGS) has emerged as a robust diagnostic tool for this condition. This study aims to evaluate the detection efficiencies of different exome-based sequencing techniques.

**Methods:**

Exome-based epilepsy panel tests, clinical exome sequencing (CES), and whole exome sequencing (WES) were conducted on 259 pediatric patients diagnosed with epilepsy. Single-nucleotide variants (SNVs) and copy number variants (CNVs) were interpreted based on each patient’s phenotypic presentation. Additionally, data concerning clinical symptoms, neuroimaging findings, treatment responses, and prognostic outcomes were collected and analyzed.

**Results:**

The overall diagnostic yield was 32.8% (85/259), with a diagnostic yield of 40.0% for exome-based epilepsy panels, 30.1% for CES, and 27.8% for WES. We identified 82 cases with pathogenic or likely pathogenic SNVs and 4 cases with pathogenic CNVs, of which one case with both SNV and CNV. The most frequently detected gene was PRRT2, present in 10.0% (9/82) of cases. Epileptic syndromes were diagnosed in 66 patients, predominantly West Syndrome, Dravet Syndrome and Genetic Epilepsy with Febrile Seizures plus.

**Conclusion:**

NGS is an effective method for uncovering the genetic foundations of pediatric epilepsy, with diagnostic yields varying based on the sequencing approach used. The growing preference for WES underscores its utility in complex cases, pointing to a trend towards more tailored diagnostic strategies.

## 1 Introduction

Epilepsy is a common neurological disorder in pediatrics characterized by a highly variable phenotype. Clinical manifestations range from self-limiting seizures to severe epileptic encephalopathy. The causes of epilepsy are diverse, including genetic abnormalities, structural changes, metabolic disorders, immune dysfunctions, and unknown etiologies (Scheffer et al., 2017). Genetic factors are responsible for 70%–80% of cases (Myers and Mefford, 2015a).

The genetic landscape of epilepsy is intricate, comprising CNVs, SNVs, small insertions or deletions (indels), and dynamic variations. There is significant phenotypic overlap among variants in various epilepsy-associated genes. The deployment of NGS has markedly enhanced the molecular diagnosis of epilepsy, facilitating rapid identification of causative genes, which supports prognosis assessment and the implementation of precision medicine. The clinical utility of NGS has been validated in conditions such as epileptic encephalopathies (Perucca et al., 2017b; Demos et al., 2019; Yang et al., 2019) as well as neurodevelopmental disorders where epilepsy is a secondary feature (Soden et al., 2014; Thevenon et al., 2016). Several diagnostic strategies, including gene panels, whole-exome sequencing (WES), and whole-genome sequencing (WGS), are currently employed to identify the genetic underpinnings of epilepsy. Generally, the diagnostic yield improves by analyzing a broader array of genes (Mercimek-Mahmutoglu et al., 2015; Mei et al., 2017).

In recent years, our department has adopted several diagnostic procedures for epilepsy, influenced by an evolving understanding of its genetic foundations and a reduction in the costs of high-throughput sequencing technologies. This study not only evaluates the efficacy and indications of various diagnostic approaches but also analyzes shifts in the trends of genetic testing strategies over time.

## 2 Materials and methods

### 2.1 Patients

A total of 259 Han Chinese pediatric patients from the Department of Neurology, Shenzhen Children’s Hospital were retrospectively studied. The inclusion criteria were: 1) clinically diagnosed with epilepsy by pediatric neurologists following the International League Against Epilepsy criteria (2017) (Fisher et al., 2017); 2) referred by pediatric neurologists for genetic tests between August 2018 and July 2021; 3) less than 18 years old at the time of epilepsy diagnosis; 4) patients whose epilepsy was not caused by head trauma or central nervous system infections. None of the evaluated individuals had consanguinity, and none were on medication for other neurological disorders during the evaluation. The patients’ family history, clinical presentation, electroencephalogram (EEG), brain magnetic resonance imaging (MRI), therapy options, and prognosis were collected and systematically reviewed. Aside from the balance between diagnostic yield and cost-effectiveness, the selection of different genetic testing methods was primarily based on the clinical presentation and the complexity of the case. Gene panel sequencing for epilepsy was recommended for patients with clear clinical indicators suggestive of specific epilepsy syndromes. For patients with more ambiguous or complex clinical features, Clinical exome sequencing (CES) was chosen to broaden the scope of genetic analysis beyond epilepsy-specific genes. WES was employed for patients with atypical or syndromic presentations, particularly when previous genetic testing did not yield a diagnosis or when the clinical features suggested a broader or more complex genetic basis. Mitochondrial genome sequencing was recommended if the patients presented suspicious mitochondrial disorder symptoms. The study was approved by the Shenzhen Children’s Hospital ethics committee. Written informed consent was obtained from all parents or their legal guardians.

### 2.2 Whole-exome sequencing

Peripheral blood from the patients and their parents (if available) was collected for DNA extraction using the Solpure Blood DNA Kit (Magen). DNA was fragmented to 300–500 bp using the Q800R Sonicator (Qsonica). Libraries were prepared with the Agilent Sureselect Human All Exon v5 kit (Agilent Technologies, Santa Clara, CA, USA) and were sequenced on the HiSeq 2000 (Illumina, San Diego, CA, USA). The average coverage depth was about 80–100×, with over 99% of the target regions. Fast software was used to process raw data to remove adapters and filter low-quality reads. Sequencing reads were mapped to the human reference genome (GRCh37/hg19) by NextGENe software (SoftGenetics, State College, PA, USA).

### 2.3 Clinical-exome sequencing and epilepsy gene panel

Custom-designed NimbleGen SeqCap probes (Roche NimbleGen, Madison, WI, United States) were used for in-solution hybridization. DNA samples were indexed and sequenced on the AmCareSeq-2000 (Amcare, Guangzhou, China). The average coverage depth was about 200×, with over 99% of the target regions covered by at least 20 reads. The processing methods of the raw data were consistent with those used for WES. The clinical-exome sequencing targeted coding exonic regions of approximately 5000 OMIM-recorded genes, known pathogenic variants from deep intronic or other non-coding regions. Similarly, epilepsy gene panel sequencing uses the same sequencing and data processing methods as CES. Still, it included 2,881 genes compiled through a comprehensive literature search (Pubmed, OMIM) and clinically available epilepsy panels.

### 2.4 Mitochondrial genome sequencing

Mitochondrial genome sequencing was conducted by long-range PCR (LR-PCR) and subsequent deep coverage sequencing (Zhang et al., 2012). In short, Mt-DNA was first amplified by LR-PCR and disrupted using ultrasonication. Mitochondrial libraries were established using the KAPA HTP Library Preparation Kit (Kapa Biosystems, USA). Sequencing was performed on HiSeq 2000 (Illumina). The average depth of sequencing was at least 5,000×. Variations were quantified by their percentage value. The lower limit of detection of variation heteroplasmy in the coding region is 2%.

### 2.5 Variant analysis and interpretation

Nucleotide variants identified in the aligned reads were extracted and analyzed using the NextGENe software (Version 2.4.2; SoftGenetics, State College, PA, United States). Variants were annotated using population databases (gnomAD, 1,000 Genomes, dbSNP) and variants databases (Clinvar, HGMD). In silico predictions were performed using the PolyPhen-2, SIFT, PROVEAN, MutationTaster, and GeneSplicer software tools. Sanger sequencing was performed to validate the variants identified by NGS. CNVs were interpreted based on the Database of Genomic Variants (DGV, http://dgv.tcag.ca/dgv/app/home), DECIPHER (https://decipher.sanger.ac.uk/), OMIM (https://www.omim.org/). The resolution was 100 kb with a bin size of 25 kb. Variants were classified as pathogenic (P), likely pthogenic (LP), variant of uncertain significance (VUS), likely benign (LB), or benign (B) according to the ACMG guideline (Richards et al., 2015b; Riggs et al., 2020).

### 2.6 Statistical analysis

Data were analyzed using SPSS software (version 23.0; IBM SPSS Statistics, Armonk, NY). Quantitative data were expressed as mean ± standard deviation (x ± s), while categorical data were presented as numbers and percentages (n%). The chi-squared test was used to compare categorical variables, including age at onset distribution, sex distribution, epilepsy classifications, syndromic diagnoses, the occurrence of developmental delay/intellectual disability (DD/ID), and findings on brain MRI. *p*-values less than 0.05 were considered statistically significant.

## 3 Results

### 3.1 Demographics and clinical characteristics

**FIGURE 1:**
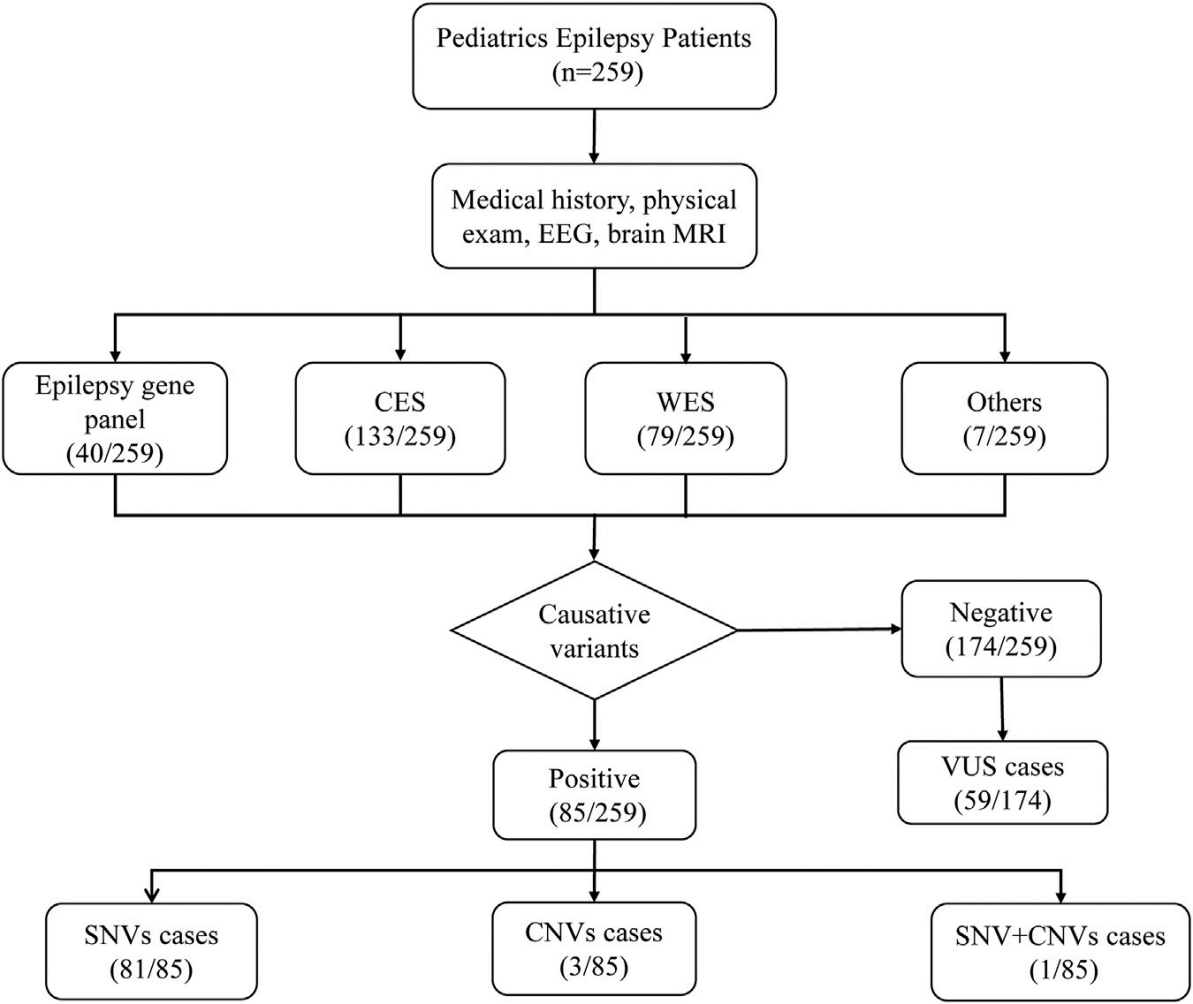
Schematic summarizing the 259 epilepsy cases.

A total of 259 Han Chinese pediatric patients with epilepsy were included, and their demographic and clinical information are shown in Table 1. The male-to-female ratio was 1.49:1. Age at epilepsy onset ranged from 2 days to 14 years old (median 18 months). Only patients detected with pathogenic/likely pathogenic variants were considered to have causative variants, while patients detected with VUS or undetected variants were not included. Patients with causative variants were more likely to have onset from 1 month to the first year of life compared to those without causative variants (57.6% vs 28.7%, *p* < 0.001). The most frequent type of epilepsy was focal epilepsy (155/259, 59.8%). There were 66 patients diagnosed with epileptic syndromes, of which the most common were West syndrome (WS) (27/259, 10.4%), followed by Dravet syndrome (DS) (10/259, 3.9%), Genetic Epilepsy with Febrile Seizures plus (GEFS+) (10/259, 3.9%), and Lennox-Gastaut syndrome (LGS) (7/259, 2.7%). Other diagnosed epileptic syndromes included Ohtahara syndrome (OS), childhood absence epilepsy (CAE), benign familial infantile epilepsy (BFIE), Doose syndrome (epilepsy with myoclonic-astatic seizures, EMAS), Panayiotopoulos syndrome (PS), juvenile myoclonic epilepsy (JME), epilepsy and mental retardation limited to females (EFMR), electrical status epilepticus during sleep (ESES), and Benign childhood epilepsy with centrotemporal spikes (BECT). In addition, 139 of the 259 patients (53.7%) combined with DD or ID. Patients with causative variants were more likely to combine with DD/ID (70.6% vs45.4%, *p* = 0.001). Brain MRI found 143 (55.2%) patients showed neurologic abnormalities. The flowchart of the sequencing results was shown in Figure 1.

**TABLE 1:** Demographics and clinical information of the patients.

Variables	Total number, n (%)	Positive patients, n (%^a^)	Negative patients, n (%^b^)	*p*
Total	259	85 (32.8%)	174 (67.2%)	
Sex				0.614
Male	155	49 (57.6%)	106 (60.9%)	
Female	104	36 (42.4%)	68 (39.1%)	
Age at onset				<0.001
0–1 month	10	3 (3.5%)	7 (4.0%)	
1 month - 1 year	99	49 (57.6%)	50 (28.7%)	
over 1 year	143	29 (34.1%)	114 (65.5%)	
Not determined	7	4 (4.7%)	3 (1.7%)	
Epilepsy classifications				0.364
Generalized epilepsy	53	16 (18.8%)	37 (21.3%)	
Focal epilepsy	155	48 (56.5%)	107 (61.5%)	
Generalized and focal epilepsy	51	21 (24.7%)	30 (17.2%)	
Epilepsy syndromes				
WS	27	7 (8.2%)	20 (11.5%)	0.402
DS	10	7 (8.2%)	3 (1.7%)	0.011
LGS	7	2 (2.4%)	5 (2.9%)	0.808
GEFS^+^	10	2 (2.4%)	8 (4.6%)	0.379
OS	2	1 (1.2%)	1 (0.6%)	0.603
CAE	2	0 (0.0%)	2 (1.1%)	0.321
EMAS	1	1 (1.2%)	0 (0.0%)	0.152
BFIE	2	2 (2.4%)	0 (0.0%)	0.042
PS	1	0 (0.0%)	1 (0.6%)	0.484
JME	1	0 (0.0%)	1 (0.6%)	0.484
EFMR	1	1 (1.2%)	0 (0.0%)	0.152
ESES	1	0 (0.0%)	1 (0.6%)	0.484
BECT	1	0 (0.0%)	1 (0.6%)	0.484
Unclassified	193	62 (72.9%)	131 (75.3%)	0.684
DD/ID				
Yes	139	60 (70.6%)	79 (45.4%)	0.001
No	101	21 (24.7%)	80 (46.0%)	
Not determined	19	4 (4.7%)	15 (8.6%)	
Brain MRI				0.797
With findings	143	46 (54.1%)	97 (55.7%)	
No findings	104	34 (40.0%)	70 (40.2%)	
Not determined	12	5 (5.9%)	7 (4.0%)	

^a^ Percentage among the 85 cases.

^b^ Percentage among 174 cases. BECT: Benign childhood epilepsy with centrotemporal spikes; BFIE: Benign familial infantile epilepsy; CAE: Childhood absence epilepsy; DD: Developmental delay; DS: Dravet syndrome; GEFS^+^: Genetic Epilepsy with Febrile Seizures plus; EMAS: Epilepsy with myoclonic-atonic seizures; EFMR: Epilepsy and mental retardation limited to females; ESES: Electrical status epilepticus during sleep; ID: Intellectual disability; JME: Juvenile myoclonic epilepsy; LGS: Lennox-Gastaut syndrome; OS: Ohtahara syndrome; PS: Panayiotopoulos syndrome; WS: West Syndrome.

#### 3.2 Application of exome-based sequencing strategies

**FIGURE 2:**
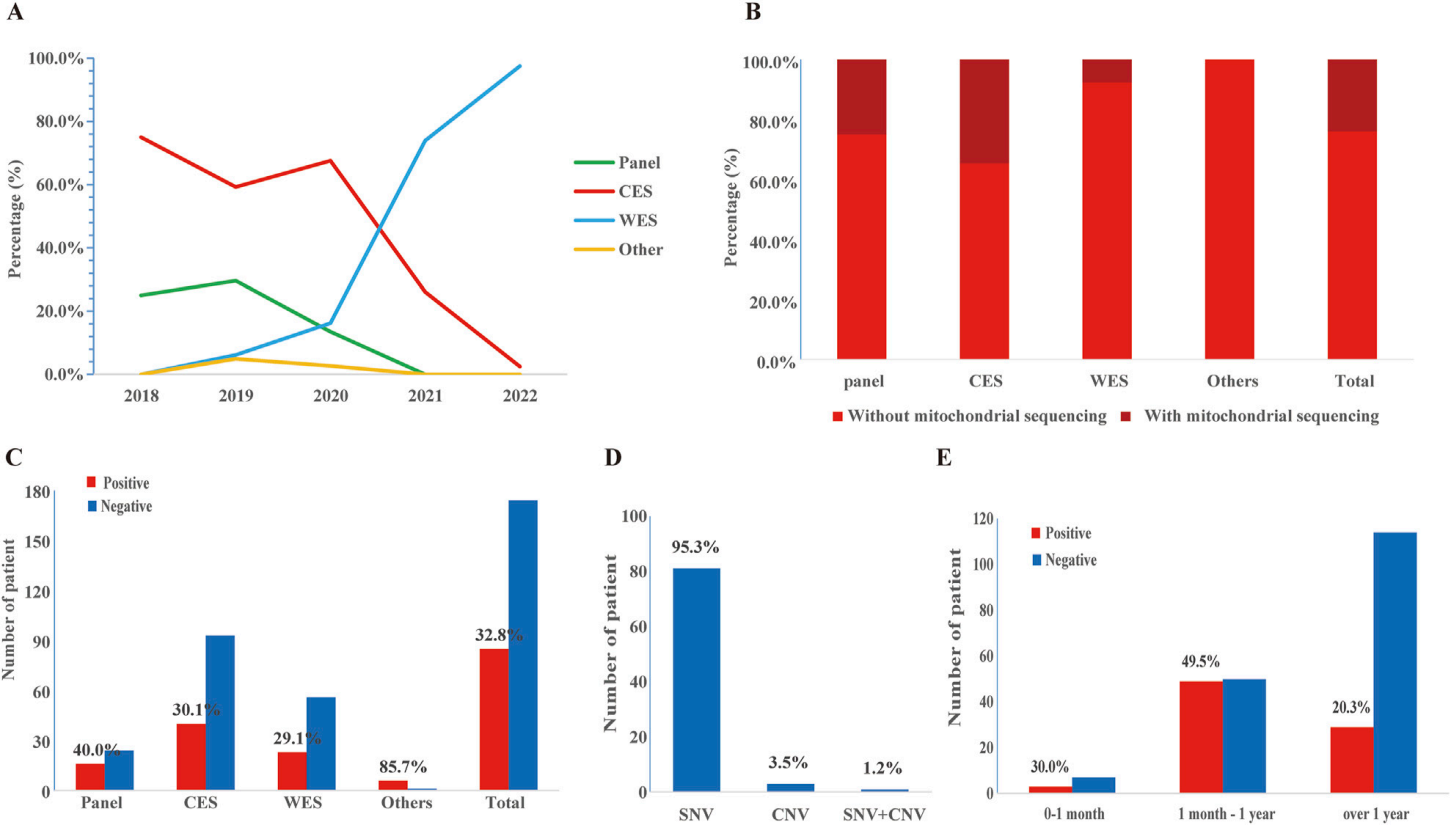
The application and diagnostic yield of different sequencing strategies. **(A)** The application of epilepsy gene panel, CES, and WES over time. **(B)** The application of mitochondrial genome sequencing. **(C)** Number of patients and diagnostic yield of epilepsy gene panel, CES, and WES. **(D)** Distribution of pathogenic/likely pathogenic (P/LP) single nucleotide variants (SNVs) and copy number variations (CNVs) in the study cohort. **(E)** Number of patients and detection rates at different ages of epilepsy onset. Detection rates were calculated as the ratio of the total number of positive cases in the age range of epilepsy onset. Additionally, please modify the sentence in Section 3.3 Diagnostic Yield (Line 377), changing “while CES (30.1%) and WES (27.8%)” to “while CES (30.1%) and WES (29.1%)”.

Overall, the majority of patients received CES (133/259, 51.4%), followed by WES (79/259, 30.5%) and epilepsy gene panel (40/259, 15.4%). However, the selection of sequencing methods had changed over the years (Figure 2A). CES (75.0%, 59.3%) constituted the majority in 2018–2019, while WES (0%, 6.2%) was the less common option. In 2020, CES retained its dominance (67.6%), but the preference for WES increased (16.2%), and the use of epilepsy gene panels decreased. In 2021–2022, WES became the preferred method (73.9%, 97.5%), surpassing CES (26.1%, 2.5%), with epilepsy gene panels being rarely chosen. Additionally, 62 of 259 cases conducted mitochondrial genome sequencing spontaneously (Figure 2B).

#### 3.3 Diagnostic yield

A molecular diagnosis was established in 85 of 259 patients, resulting in a comprehensive diagnostic yield of 32.8% (Table 1). The diagnostic yield of each method was shown in Figure 2C. Surprisingly, the epilepsy gene panel had the highest diagnostic yield (40.0%), while CES (30.1%) and WES (27.8%) yielded similar results. As shown in Figure 2D, P/LP SNVs were found in 81 cases (95.3%) (including 3 cases with mitochondrial DNA (mt-DNA) variants), P/LP CNVs were found in 3 cases (3.5%), and concomitant likely pathogenic SNV and CNV were found in 1 case (1.2%). In addition, variants of uncertain significance possibly explaining the epilepsy of the patient were identified in 59 cases (59/259, 22.8%), which included 57 SNVs and 2 CNVs (Supplementary Table S1).

Patients who started epilepsy at 1 month to 1 year old had more P/LP variants (49.5%) than those onsets after 1 year old (20.3%) and before 1 month (30.0%) (Figure 2E). The molecular diagnostic rates in different clinical syndromes and their relevant genes were shown in Table 2. Among the 259 patients, West syndrome was clinically diagnosed in 27, seven of which were molecular diagnosed (detection rate: 25.9%). One SNV was found in *NUS1*, *BRAF*, *CDKL5*, *IRF2BPL*, *SCN8A*, *COQ4*, and *KCNT1,* respectively. 22q11.21 duplication was found in the same case that had a *COQ4* variant. DS was clinically diagnosed in 10 cases, seven of which were molecular diagnosed (detection rate as 70.0%). Seven distinct SNVs were identified in *SCN1A*. Genetic Epilepsy with febrile seizures plus was clinically diagnosed in 10 cases, 2 of which were molecular diagnoses (detection rate as 20.0%). One of each SNV was found in SCN1A and KMT2D. Lennox-Gastaut syndrome was clinically diagnosed in 7 cases, two of which were molecularly diagnosed. (Detection rate as 28.6%). One of each SNV was found in *ADSL* and *TSC2*. Tuberous sclerosis was clinically diagnosed in 5 cases, and all of them were molecular diagnosed (detection rate as 100%). Other clinical syndromes and their related genes were listed in Table 2.

**TABLE 2:** Clinically diagnosed syndrome and associated genes.

Clinically diagnosed syndrome	Diagnostic yield	Genes
WS	7/27 (25.9%)	*NUS1* (1)*, BRAF* (1)*, CDKL5* (1)*, IRF2BPL* (1)*, SCN8A* (1), (*COQ4,* 22q11.21) (1), *KCNT1* (1)
DS	7/10 (70.0%)	*SCN1A* (7)
GEFS^+^	2/10 (20.0%)	*SCN1A* (1)*, KMT2D* (1)
LGS	2/7 (28.6%)	*ADSL* (1), *TSC2*(1)
TSC	5/5 (100%)	*TSC1* (1), *TSC2* (4)
OS	1/2 (50.0%)	*GALC* (1)
BFIE	2/2 (100.0%)	*PRRT2* (2)
EMAS	1/1 (100%)	*SLC6A1* (1)
EFMR	1/1 (100%)	*PCDH19* (1)
RS	1/1 (100%)	*MECP2* (1)

FootNote: BFIE: Benign familial infantile epilepsy; DS: Dravet syndrome; GEFS^+^: Genetic Epilepsy with Febrile Seizures plus; EFMR: Epilepsy and mental retardation limited to females; EMAS: Epilepsy with myoclonic-atonic seizures; LGS: Lennox-Gastaut syndrome; OS: Ohtahara syndrome; TSC: Tuberous sclerosis; RS: Rett syndrome; WS: West Syndrome.

#### 3.4 Detection of single-nucleotide and copy-number variants

**FIGURE 3:**
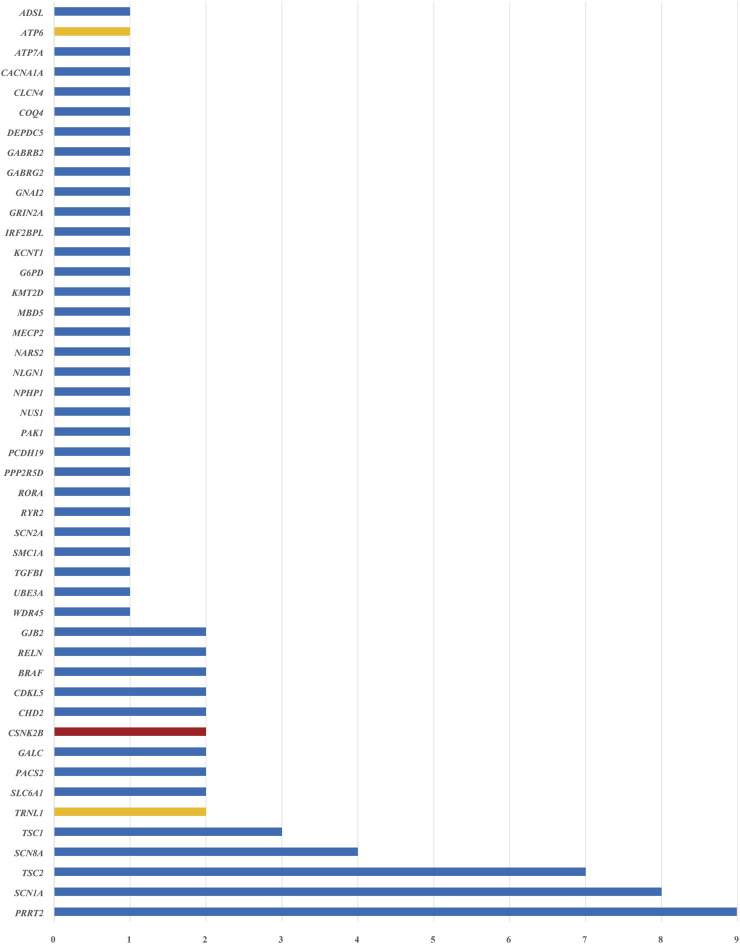
Disease-related genes were identified in 259 patients with epilepsy. The blue bars indicate nuclear genes, the orange bars indicate mitochondrial genes, and the red bar indicates a gene that was not included in the epilepsy gene panel. The horizontal axis represents the number of patients with pathogenic or likely pathogenic SNVs identified for each gene.

**FIGURE 4:**
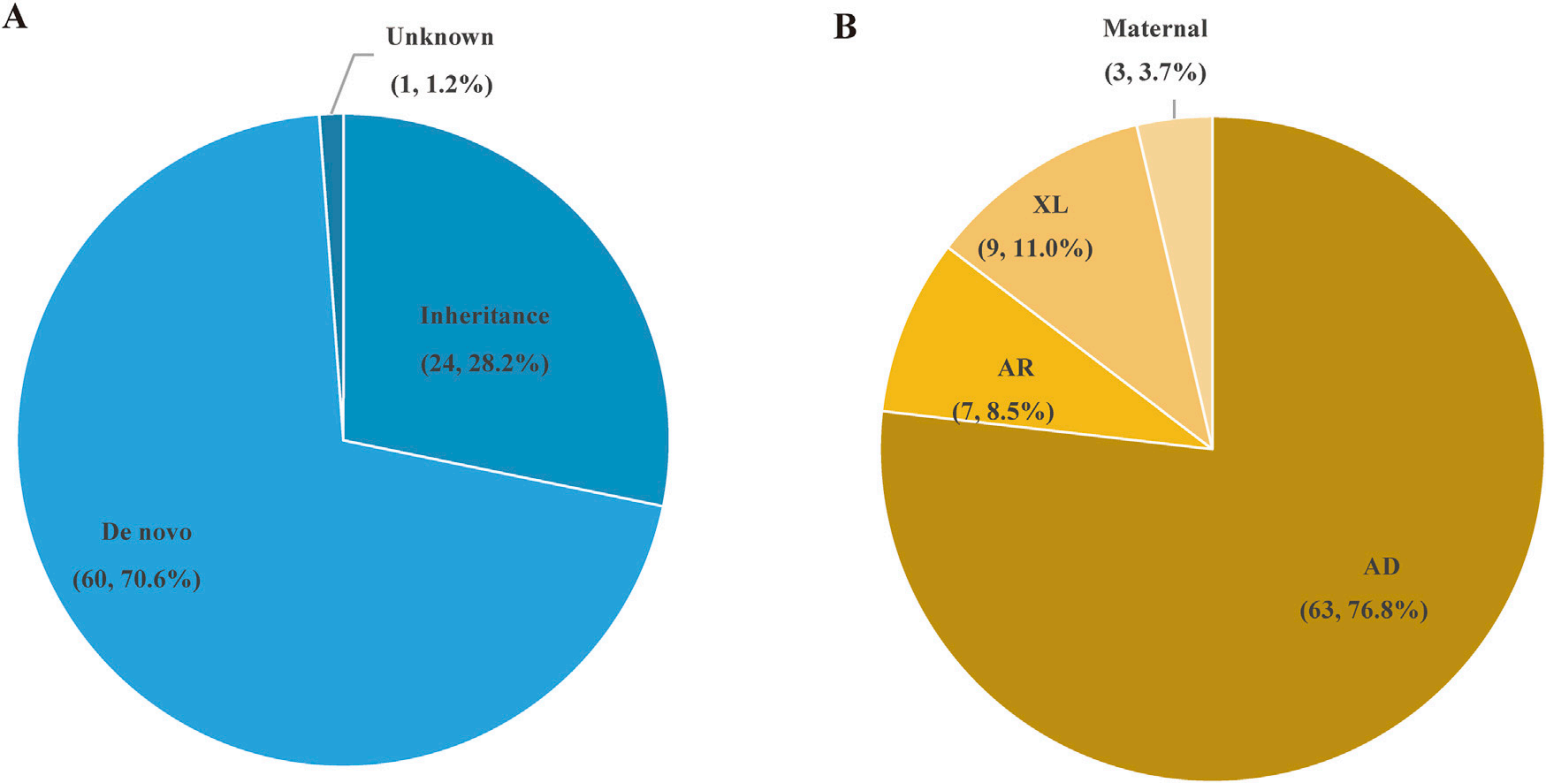
The types of variation and inheritance patterns in 84 P/LP patients with epilepsy. **(A)** Distribution of de novo and inherited genetic variants in the patient cohort. **(B)** Inheritance patterns of the genetic variants, including autosomal dominant (AD), autosomal recessive (AR), X-linked (XL), and maternal inheritance.

A total of 85 SNVs were identified in 82 cases, assigned to 44 nuclear genes and 2 mt-DNA genes (Supplementary Table S2). The most frequent nuclear gene was *PRRT2* (9, 10.0%), followed by *SCN1A* (8, 9.8%) and *TSC2* (7, 8.5%), *SCN8A* (4, 4.9%), *TSC1* (3, 3.7%) (Figure 3). Two patients (2.4%) had variants in *SLC6A1, PACS2, GALC, CSNK2B, CHD2, CDKL5, BRAF, RELN,* and *GJB2,* respectively. Three SNVs in 2 mitochondrial genes, *TRNL1* and *ATP6,* were discovered in 3 cases, respectively. According to the OMIM database, 63 variants (76.8%) were inherited in autosomal dominant mode, 9 variants (11.0%) were inherited in X-linked mode, seven variants (8.5%) were inherited in autosomal recessive mode, and 3 mitochondrial variants (3.7%) were maternally inherited. The origin of the variants was confirmed by trio-based sequencing or Sanger sequencing. Sixty of the SNVs were *de novo* (70.6%), and only 24 (28.2%) SNVs were inherited from the parents. (Figure 4).

Four pathogenic/likely pathogenic CNVs were identified in 4 cases (4/259, 1.5%) (Supplementary Table S3). The length ranged from 398 kb to 5.56Mb, with 3 deletions and 1 duplication. The CNVs were located in chromosomes 1, 15, 16, and 22, respectively, and all were *de novo*. These CNVs contained or partially contained epilepsy-related genes, such as *PRRT2* (16p11.2), *ZBTB18* (1q43q44), and *GABRB3* (15q11.2q13.1).

Interestingly, patient 133 presented ocular hypertelorism, brain hypoplasia, West syndrome, global developmental delay, and co-enzyme Q10 deficiency. CES detected likely pathogenic homozygous variants c.370G > A (p.G124S) in the *COQ4* gene related to Leigh syndrome. Another likely pathogenic CNV was also found in the patient, 1.32 Mb duplication of 22q11.21, which contains 27 genes recorded in OMIM. The variants in *COQ4* could explain epilepsy, brain hypoplasia, and DD. The duplicated region contained *TBX1*, which is related to DiGeorge syndrome or Velocardiofacial syndrome. The previously reported cases with 22q11.21 duplication syndrome showed DD, face anomaly, hearing damage, and congenital heart disease (Firth, 1993). Therefore, it is likely that both SNV and CNV influenced the patient’s phenotypes.

### 4 Discussion

In clinical practice, NGS has been demonstrated to be a useful diagnostic tool in diagnosing the cause of epilepsy. The identification of causative variants enables improvements in treatment options for patients. Our results revealed P/LP variants in 44 nuclear genes, with *PRRT2*, *SCN1A*, and *TSC2* being the top three most frequently mutated, collectively accounting for 28.3% of all P/LP variants. The frequency of *PRRT2* variants (10.0%) in this cohort, predominantly associated with BFIE, is notably higher than the 2%–6% reported in Western populations (Landolfi et al., 2021). *PRRT2* plays a critical role in synaptic transmission by interacting with SNAP25 in the SNARE complex, a pathway essential for neurotransmitter release (Lee et al., 2012). Disruption of *PRRT2* function may alter synaptic dynamics, contributing to seizure susceptibility. The strong familial inheritance pattern observed in *PRRT2*-related cases (77.8%) highlights the importance of integrating family history into genetic evaluations. The hotspot *variation* c.649dupC (p.R217Pfs*8), identified in six cases, further supports its role as a diagnostic marker for BFIE, with prior studies reporting this variant in 50%–70% of *PRRT2*-related epilepsy cases globally (Steinlein et al., 2012). *SCN1A*, present in 9.8% of cases, was primarily associated with DS, aligning with prior studies indicating frequencies ranging from 8%–15% in epilepsy cohorts (Zuberi et al., 2011; Olson et al., 2017; Yang et al., 2019). *SCN1A* encodes the Nav1.1 voltage-gated sodium channel ɑ-subunit, which is critical for maintaining inhibitory signaling in GABAergic neurons. Variations in *SCN1A* disrupt sodium channel function, impairing GABAergic inhibitory neurotransmission and leading to hyperexcitability, a hallmark of early-onset epilepsy (Catterall et al., 2010; Brunklaus et al., 2012). Notably, 87.5% of *SCN1A* variants in our cohort occurred *de novo*, consistent with global findings that 80%–90% of *SCN1A variation*s arise *de novo* in DS patients (Depienne et al., 2009; Scheffer and Nabbout, 2019). Variability in seizure phenotypes among *SCN1A*-mutated patients suggests potential interactions with other ion channel genes, such as *SCN2A* and *SCN8A*, underscoring the polygenic complexity of *SCN1A*-related epilepsies (Lossin, 2009; Howell et al., 2015). *TSC2* variations were identified in 8.5% of patients and were predominantly linked to earlier epilepsy onset and a higher prevalence of DD/ID, consistent with prior studies reporting *TSC2* variations in 6%–10% of pediatric epilepsy cohorts (Crino, 2011).

Besides, TSC2, a critical component of the TSC1/TSC2 complex, regulates mTORC1 signaling, essential for synaptic plasticity, neural development, and cellular growth. Loss of *TSC2* function leads to hyperactivation of mTOR, resulting in aberrant neural development and epileptogenesis. Patients with *TSC2* variations may benefit from mTOR inhibitors like everolimus, which have demonstrated a >50% reduction in seizure frequency in TSC-related epilepsy patients (Krueger et al., 2013). Additionally, *SCN1A*-related network instability could exacerbate mTOR dysregulation, enhancing epileptogenesis (Howell et al., 2015). Similarly, *PRRT2* disruptions, through their role in synaptic vesicle release, may indirectly impact mTOR signaling, highlighting a convergent mechanism linking ion channel dysfunction, synaptic regulation, and mTOR pathways. Variants in *CSNK2B* (OMIM: 115441) were identified in 2 patients through WES (Case 67 and 72), but this gene was not included in our epilepsy gene panel. *CSNK2B* causes Poirier-Bienvenu neurodevelopmental syndrome, which is characterized by early-onset epilepsy (median 5 months), clustered GTCS, myoclonic seizures, and DD (Bonanni et al., 2021). In addition, mt-DNA variants are also considered responsible for epilepsy and other neurological diseases (Rahman, 2012). We performed mitochondrial genome sequencing on 62 patients and detected 3 mt-DNA variants (3/259,1.2%). Each m.3243A > G variant was detected in case 78 and 96, respectively, which is a widespread variant in mitochondrial t-RNA and related to MELAS syndrome (Tanahashi et al., 2000). In case 136, another mt-DNA variant, m.8993T > G (p.L156R), was found in *ATP6*, which is related to Leigh syndrome (Ganetzky et al., 2019).

Additionally, 59 VUS were identified in this study, representing 22.8% of the cohort. Although VUS currently do not directly influence clinical decision-making, advances in functional studies, expanding genomic databases, and improved genotype-phenotype correlation frameworks could facilitated the reclassification of VUS over time. This reanalysis has the potential to improve diagnostic accuracy, support genetic counseling, and enable more precise treatment strategies (Richards et al., 2015a; Lindy et al., 2018). It is worth noting that there were five variations in *CACNA1H*. According to the ClinGen Epilepsy Gene Curation Expert Panel, *CACNA1H* is classified as a disputed gene due to insufficient evidence supporting its role as a monogenic cause of epilepsy (Helbig et al., 2018). While *CACNA1H* encodes a T-type calcium channel crucial for neuronal excitability, studies suggest its variants often act as susceptibility factors in polygenic or multifactorial contexts rather than as definitive monogenic causes (Weiss and Zamponi, 2020). Despite this, *CACNA1H* variants were included in this analysis to provide a comprehensive overview of all identified variants, given their potential role in seizure susceptibility and frequent detection in epilepsy cohorts. These findings should be interpreted cautiously in light of the gene’s uncertain clinical relevance as a monogenic cause. This also underscored the importance of refining gene classifications to clarify the precise role of *CACNA1H* in epilepsy through further functional studies and larger cohort analyses.

CNVs are a well-known genetic cause of epileptic syndromes and other neurological disorders. Our study established that the detection rate of CNVs was 1.5%, which was relatively lower than that of the previous studies (Jiang et al., 2021; Zou et al., 2021). The possible explanation is that CMA/CNV-seq is the first-tier diagnostic tool for CNV detection. If the patients detected P/LP CNVs that can explain their epilepsy through CMA/CNV-seq, they would not undergo additional genetic tests unless they were suspected of monogenic diseases.

Many prior cohort studies examined the effectiveness of various sequencing strategies in pediatric epileptic patients, with diagnostic yields ranging from 26.7% to 42% (Jang et al., 2019; Yang et al., 2019; Rochtus et al., 2020b; Chen et al., 2021). Gene selection principles have significant variability in different exome-based epilepsy gene panels, which range in size from 17 to 2,742 genes (Zhang et al., 2017; Yang et al., 2019). Mercimek-Mahmutoglu et al. (Mercimek-Mahmutoglu et al., 2015) reported an increasing diagnostic yield, from 10% to 48.5%, when the number of genes expanded from 35 to 265. However, the diagnostic yield did not always associate with a more significant number of genes. Kim et al. (Kim et al., 2021a) suggested an overall diagnostic yield to be 47.3% using an epilepsy gene panel containing 79–127 genes, while Chen et al. (Chen et al., 2021) achieved a diagnostic yield of 32.7% using WES. Similar results were seen in our study as well: We found that the epilepsy gene panel (40.0%) had a higher diagnostic yield than CES (30.1%) and WES (29.1%). A previous study in our department performed whole-genome sequencing (WGS) on 320 children with epilepsy. The overall diagnostic yield of WGS was 36.6% (Zou et al., 2021), comparable to our study (32.8%). The possible explanation could be that in earlier years, limited by the understanding of epilepsy genes and the cost considerations, genetic testing was restricted to patients with specific indications such as distinctive dysmorphic features, known family history, or indicative laboratory results. Subsequently, due to the increasing diagnostic needs and cost reduction, CES and WES were performed on more patients with non-specific phenotypes. Reducing diagnostic yield may be caused by the growing number of non-specific patients who received genetic tests. The preference for WES over epilepsy gene panels has grown over time.

Furthermore, our analysis revealed that the diagnostic yield varied significantly across patient subgroups, highlighting the interplay between epilepsy phenotypes and their genetic underpinnings. Patients diagnosed between 1 month and 1 year of age showed the highest diagnostic yield, which underscores the importance of early genetic testing in suspected genetic epilepsy syndromes (Kim et al., 2021a). This finding aligns with previous studies indicating that early-onset epilepsies are more likely to have a genetic basis (Rochtus et al., 2020b; Jiang et al., 2021). These aspects emphasize the need for a personalized approach in the genetic testing of pediatric epilepsy, taking into account the specific clinical presentation. Among the 139 cases (53.7%) with epilepsy accompanied by DD/ID, the diagnostic yield was significantly higher (70.6%) compared to patients without DD/ID (24.7%). These findings emphasize the distinct genetic basis of epilepsy with neurodevelopmental comorbidities, where pathogenic variants frequently contribute to both conditions. Genes like *SCN1A* and *TSC2*, identified in this study, are associated with syndromes like DS and tuberous sclerosis complex, both featuring epilepsy and developmental impairments. These results are consistent with prior research showing diagnostic yields of 60%–80% in similar cohorts, particularly when early-onset epilepsy and neurodevelopmental symptoms coexist (Lindy et al., 2018; Rochtus et al., 2020a). The co-occurrence of epilepsy with DD/ID provides phenotypic clarity, which aids in the interpretation of variants and increases the likelihood of identifying monogenic causes. Neurodevelopmental comorbidities often involve variations in pleiotropic genes, further underscoring the need for comprehensive genomic approaches (Myers and Mefford, 2015b). Focal epilepsies demonstrated a significantly higher diagnostic yield (56.5%) compared to generalized epilepsies (18.8%). This result, consistent with prior studies, reflects the relatively well-defined genetic basis of focal epilepsies, often involving single-gene variations in genes like *SCN1A* (Lindy et al., 2018; Kim et al., 2021b). In contrast, generalized epilepsies showed a more complex genetic architecture, including polygenic contributions and gene-environment interactions, which are less detectable with current sequencing platforms (Myers and Mefford, 2015b; Perucca et al., 2017a). Focal epilepsies also present with more specific clinical features, such as seizure semiology and focal imaging findings, enhancing the accuracy of variant interpretation. In comparison, the broader and overlapping phenotypes of generalized epilepsy complicate genetic analysis and reduce diagnostic yields. Moreover, the design of gene panels and exome approaches frequently prioritizes genes associated with focal epilepsy syndromes, potentially biasing diagnostic outcomes toward these phenotypes (Rochtus et al., 2020b). These findings collectively highlight the importance of integrating early and comprehensive genetic testing, tailored to the clinical presentation, to improve diagnostic outcomes across diverse epilepsy subtypes.

Our study elucidates the diagnostic utility of exome-based sequencing in pediatric epilepsy. While treatment and follow-up were conducted, the assessments during the follow-up did not systematically evaluate changes in the epilepsy syndromes. This limits our understanding of the dynamic and evolving nature of epilepsy and how it responds to treatment over time. Future research should include structured and periodic evaluations of epilepsy syndromes during follow-ups to capture potential changes in the epilepsy syndromes. Additionally, the sample size for this study was relatively small, which limits the generalizability of the findings. Efforts will be made in future studies to recruit a larger cohort, providing a deeper understanding of the utility of exome sequencing in pediatric epilepsy.

### 5 Conclusion

In summary, our study underscores the significant utility of exome-based sequencing in diagnosing pediatric epilepsy, revealing distinct advantages and evolving preferences among different sequencing methods. We observed a notable shift from targeted epilepsy gene panels, which provided the highest diagnostic yields, to whole-exome sequencing. This transition reflects WES’s broader diagnostic scope, capturing a more comprehensive array of genetic abnormalities beyond those traditionally associated with epilepsy. Notably, the diagnostic yield of patients from 1 month to 1 year old was the highest, emphasizing the critical role of early genetic testing in suspected genetic epilepsy syndromes. These findings advocate for a personalized approach to genetic testing in pediatric epilepsy, tailoring the sequencing method to specific clinical presentations and the age of onset. Future studies should continue to adapt and refine genetic testing strategies, accommodating the rapid evolution of sequencing technologies to maximize diagnostic yield and inform targeted treatment strategies.

## Data Availability

The original contributions presented in the study are publicly available. This data can be found here: [https://pan.baidu.com/s/1f1shHg7ruJuQPlUnXLpYtQ?pwd=da49].
